# Does cognitive decline influence signing?

**DOI:** 10.1007/s40520-023-02523-7

**Published:** 2023-09-03

**Authors:** Alice Naomi Preti, Lorenzo Diana, Rita Castaldo, Francesca Pischedda, Teresa Difonzo, Giorgio Fumagalli, Andrea Arighi, Giuseppe Sartori, Stefano Zago, Nadia Bolognini

**Affiliations:** 1 School of Medicine and Surgery, PhD Program in Neuroscience, University of Milano-Bicocca, Monza, Italy; 2 Neuropsychology Laboratory, Department of Neurorehabilitation Sciences, IRCCS Istituto Auxologico Italiano, Milan, Italy; 3 Neurology Unit, Foundation IRCCS Ca’ Granda Hospital Maggiore Policlinico, Milan, Italy; 4 Center for Mind/Brain Sciences-CIMeC, University of Trento, Rovereto, Italy; 5 Department of General Psychology, University of Padova, Padua, Italy; 6 Department of Psychology, University of Milano-Bicocca, Milan, Italy

**Keywords:** Signature, Handwriting, Neurodegenerative, Alzheimer’s disease, Frontotemporal dementia

## Abstract

**Objective:**

The study explored the change in handwritten signature in neurodegenerative diseases by using of a rater-based approach.

**Methods:**

Four independent observers were required to compare a pair of signatures (on average, 5 years elapsed between the two signatures) made by 103 patients (mean age 72 years) with Alzheimer’s disease (AD) or frontotemporal dementia (FTD) and by 31 healthy participants (HC; mean age 73 years), judging their change according to a 0–1 rating scale (0 = similar or 1 = different). If a signature change was detected, the rater had also to report which signature features (spatial layout, omitted/added/switched letters or names, shape of letter, pen-flow) changed on the same 0–1 scale. For the AD and FTD groups, one signature was collected prior to the diagnosis of dementia, the other subsequent.

**Results:**

A signature change was reported by raters in 36% of AD patients, 44% of FTD, and 17% of HC, with significant differences between both clinical groups and HC (*vs*. AD, p = .01; *vs.* FTD, p = .001). There was not a distinctive marker of the signature change (i.e., feature change) in patients with dementia. Moreover, the signature changes in neurological patients were unrelated to their clinical and demographic characteristics (age, sex, education, time elapsed between the two signatures, Mini-mental State Examination score).

**Conclusion:**

The findings suggest a resistance of handwritten signature in neurodegenerative diseases and in physiological aging, also suggesting that the signature may be an unreliable indicator of the cognitive status in AD and FTD, at least if subjectively evaluated.

## Introduction

Handwritten signature represents a graphomotor act aimed at ‘putting a seal’ on a variety of documents, to authenticate and validate the content. Whether in its extended form (*e.g.,* on identity or legal documents) or abbreviated form (*e.g.,* the use of initials or abbreviation in more informal context), the signature, compared to spontaneous writing, develops as a faster and automated graphic gesture, as the sequence of motor commands is acquired and reproduced as a single motor unit [1, 2]. The frequent repetition of the signature throughout life indeed promotes a highly automated representation that may even be resistant to cognitive decline [1, 3–6], even when in the case of dementia [1]. This representation may not include the whole signature, but only some specific regions of it – i.e., the ones learned better and thus more automatic to be executed [2]. Recent evidence shows that signature features (e.g., stroke duration, velocity, pen pressure, etc.) seem not to differ between patients with Alzheimer’s disease at a moderate stage of illness and age-matched neurologically healthy subjects [5–7]. Eventually, the signature would be modified only in later stages of dementia, in the presence of delirium or in the case of motor disorders affecting the upper limb [8–10]. By contrast, spontaneous writing degrades during Alzheimer’s disease (AD) and frontotemporal dementia (FTD) progression, with impairment already present in the early stages of illness [11–14].

Some studies have attempted to retrospectively analyse handwriting as a gauge of cognitive status in the context of posthumous evaluation of testamentary capacity [15, 16]. Fontana et al. [15] have developed a ‘writing score’ based on the evaluation of both verbal/lexical aspects and the spatial orientation of the written test. A strong correlation emerged between such writing score and two measures of cognitive functioning, namely the Mini-Mental State Examination (MMSE, [17]) and the Milan Overall Deterioration Assessment [18] thus suggesting the possibility of retrospectively inferring a cognitive deficit from the writing even of an individual who is no longer alive. Balestrino and colleagues [16] took a step further, by judging handwriting abilities by also taking into account spelling errors in the handwritten text, along the above-mentioned writing score, proving again that an altered spontaneous writing reflects an impaired cognitive functioning.

Along the same lines, Renier et al. [5] explored the relation between writing and cognitive functioning in patients with a cognitive impairment, taking into consideration for the first time signing: neither spontaneous writing nor the level of cognitive functioning (i.e., MMSE score) were found to correlate with the signing ability of patients with a diagnosis of mild cognitive impairment (MCI) or of dementia. The authors conclude that the signing ability is independent of the cognitive status and thus it represents an unreliable indicator of a pathological cognitive decline. However, it is quite common that in forensic contexts, especially for testamentary capacity assessment, conclusions on cognitive status are drawn based on the signing ability [10]; in contrast, in clinical setting, ability to sign is never evaluated, despite its potential importance for patients’ informed consent to treatments.

Other few studies have systematically addressed the issue of the handwritten signature changes in neurodegenerative diseases, reaching similar conclusions as Renier’s [5]. For instance, Caligiuri & Mohammed [7] have shown the stability of signature features during cognitive decline, concluding that the cognitive-motor changes typical of pathological aging seem not significantly impact the signing ability. Fernandes et al. [19], by means of the kinematic analysis, have investigated the impact of AD on signature’s motor features, showing those as not altered in the initial and mid stages of AD. Finally, Pirlo and co-workers [20] have postulated the possibility to predict the development of neurodegenerative disorders by means of the analysis of signature parameters through the application of the Sigma-Lognormal model, which is based on the representation of motor commands and temporal properties of the involved movements in the act of signing. To date, no empirical evidence for this has been provided.

In light of these premises, the present retrospective study aims at deepening the evolution of signature in patients with AD or FTD by adopting a modified version of the rater-based approach originally developed by Renier and colleagues [5]. Signature changes in dementias were compared to those of neurologically healthy persons. In particular, global signature changes, and their features, were explored in AD and FTD, in order to investigate whether the signing ability: (1) changes along with cognitive decline, (2) may be informative about the level of cognitive functioning, (3) and thus could represent a cognitive marker of dementias.

## Materials and methods

### Participants

A sample of 103 participants was retrospectively recruited (60 females; mean age = 72.2 years ± 6.36 of Standard Deviation, range = 42–84 years; mean education = 9.67 ± 4.45 years, range = 3–18 years; see Table 1 for details). All participants had undergone neurological and neuropsychological evaluation at the Neurodegenerative Diseases Unit of the Fondazione IRCCS Ca’ Granda Ospedale Maggiore Policlinico in Milan (Italy), in the time span ranging from 2016 to 2019 (in accordance with the accessible data in the reference hospital unit).

**Table 1:** Demographics and clinical features of clinical groups and healthy controls

Group	N	Age (years)	M/F	Education (years)	MMSE (raw score)	Time between the signatures (years)
AD	54	73.8 ± 6.4	15/39	9.3 ± 4.3	22.2 ± 4.8	5.5 ± 3.3
FTD	49	70.4 ± 5.8	28/21	10.1 ± 4.6	20.02 ± 5.5	5.9 ± 2.5
HC	31	73.1 ± 10.7	14/17	10.6 ± 4.7	28.76 ± 1.9	4.03 ± 2.98

*AD* Alzheimer’s dementia; *FTD* frontotemporal dementia; *HC* healthy controls; *M* male; *F* female; *MMSE* Mini-Mental State Examination. For Age, Education, MMSE and the time from signature, the mean and the standard deviation are reported

Patients were included in the present study only if:

- They had a diagnosis of AD (n = 54) or FTD (n = 49) established according to multiparametric criteria: the International Working Group 2 (IWG-2) Criteria for Alzheimer’s Disease Diagnosis [21] for diagnosis of AD, the revised diagnostic criteria [22, 23] for the diagnosis of FTD;
- They had no clinical history of visual, auditory, language and motor disorder, nor muscular rigidity and hyper/hypotrophy.

All participants were right-handed, except for one left-handed in the FTD group. No patient with FTD had tremor in the dominant hand; for 2 AD patients, a minor note of a mild tremor of the dominant hand was found in the medical record.

A control group comprising 31 neurologically healthy controls (HC) was also recruited (17 females; mean age = 73.1 ± 10.7 years, range = 51–95 years; mean education = 10.6 ± 4.69 years, range = 3–18 years). HC’s inclusion criteria were a negative history of neurological and/or psychiatric diseases and absence of motor or sensory disturbances. The MMSE [17] was administered to all HC participants.

The study received ethical approval by the Committees of the *IRCCS Istituto Auxologico Italiano*, Milano (protocol n. 2021_05_18_08). The study was pre-registered on OSF Registries (https://doi.org/10.17605/OSF.IO/PRQB9).

### Signature collection and evaluation

From the hospital clinical records, demographic (i.e., age, sex, education), diagnostic (i.e., date and type of diagnosis, cerebrospinal fluid – CSF—examination), and psychometric information (i.e., the MMSE score [17]) were derived for AD and FTD participants.

Two signatures for each patient were collected: (1) one from the identity card, dating back from a pre-diagnostic period, and (2) the other, following the diagnosis, was derived from the informed-consent document for CSF examination. Only signatures at least 1 year apart were considered (mean distance in time = 5.7 ± 2.3 years, range = 1–18 years), in order to detect the evolution of the signature changes along the disease progression.

In the same way, HC were asked to provide two signatures (mean distance in time = 4.03 ± 2.98 years, range = 1–13 years): one was taken from their identity card, and the other was collected in presence, asking them to sign on an A4 sheet.

**Fig. 1:**
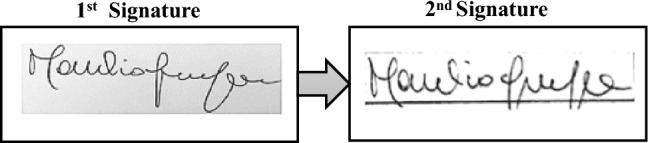
An example of a participant’s signature pair

According to Renier’s method [5], four independent raters, specifically two certified neuropsychologists and two certified neurologists, were required to compare each pair of signatures (see Fig. 1) and to rate them according to the following criteria:

1. whether they perceive a change between the two signatures: each rater was asked to judge whether the signature had changed from the first one of the ID card, by assigning either 0 (= very similar signatures) or 1 (= different signatures);
2. if a change was reported, the evaluator had to indicate which signature features had changed on the same 0–1 scale, considering the following aspects: a) the spatial layout, b) omission and/or addition and/or exchange of letters, c) omission and/or addition and/or exchange of names, d) changes of the shape of letters, and e) changes of the pen-flow (see Table 2 for details). Hence, a score of 0 indexed an unchanged signature feature, a score of 1 a changed signature.

**Table 2:** The scoring protocol for evaluating the pairs of signatures

Comparison between the signatures. How does the second signature look compared to the first?
0 = Very similar
1 = Different

If different, which signature’s feature has changed? You can choose more than one option (0 = feature not changed, 1 = feature changed)
Spatial layout: 0 – 1
Omitted/added/switched letters: 0 – 1
Omitted/added/switched names: 0 – 1
Shape of letter: 0 – 1
Pen-flow: 0 – 1

All raters were blind regarding the diagnosis or whether the signatures belonged to HC.

### Statistical analyses

Statistical analyses were performed with Jamovi (The jamovi project. *jamovi*. Version 2.2, 2013). As signatures’ change and features scores were not to distribute normally, non-parametric tests were applied.

First, the total ‘*signature change score*’ was derived for each participant by summing the four raters’ score (each ranging from 0 to 1; i.e., total raters’ score range = 0–4) and calculating the percentage of it, hence obtaining a total signature change score ranging from 0 to 100%, where: 0% = no evaluators report a change in patient’s signature, 25% = one of 4 evaluators reports a change, 50% = two of 4 raters report a change, 75% = changes detected by 3 raters, and 100% = all 4 raters indicate a change.

Then, we compared the percentages of the ‘signature change score’ of the two clinical groups and the HC by means of Kruskal–Wallis test; whenever necessary, post-hoc analyses were performed with the Dwass-Steel-Critchlow-Fligner pairwise comparisons.

Moreover, considering the whole clinical sample (i.e., both AD and FTD patients), we looked for associations, by means of series of Spearman correlations, between the ‘signature change score’ and the following clinical and demographic factors: age, sex, education, time elapsed between the two signatures, MMSE raw score at the time of the second signature. Bonferroni correction was applied to correlation analyses (alpha = 0.05/5 = 0.01). Two partial Spearman correlations were performed in order to avoid indirect effects produced by the remaining variable: a correlation between the ‘signature change score’ and age, by controlling for the time between the two signatures, and a correlation between the ‘signature change score’ and the time elapsed between the two signatures, by controlling for age. Finally, inter-rater agreement was calculated for each group as the ratio between the number of times the observers provided the same score over the total number of patients of the group.

We then considered changes related to each signature feature (i.e., spatial layout, letters, names, shape of letters, see above for details): a ‘feature change score’ was derived for each group following the same procedure used for deriving the percentage of signature change score (i.e., ranging from 0% = no raters indicate a change in a specific signature feature to 100% = all raters indicate a change in that specific signature feature). Then, Kruskal–Wallis analyses were applied for each ‘feature change score’ with the clinical groups as between-subject factor. For both groups, Spearman correlations were calculated between the ‘feature change score’ on each signature feature and relevant clinical-demographic characteristics (*e.g*., age, sex, education, the time between the two signatures, MMSE raw score). The significance level was corrected with Bonferroni (alpha = 0.05/15 = 0.003).

## Results

**Fig. 2:**
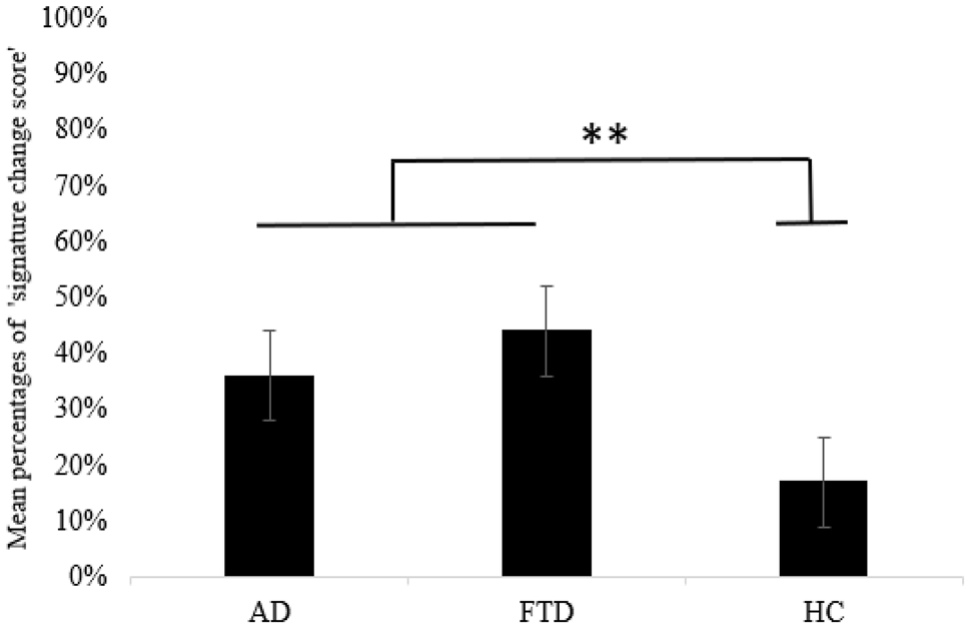
‘Signature change score’ in the experimental groups. The graph illustrates the percentage of signature change in participants with Alzheimer’s diseases (AD) or frontotemporal dementia (FTD), and in healthy controls (HC). Error bars represent the standard error of the mean; ** = significant difference between experimental groups (p < .01)

With respect to the ‘signature change score’, on average, a change was perceived by the observers in 36% of AD patients’ signatures, 44% of FTD, and 17% of HC (see Fig. 2).

Kruskal–Wallis analyses highlighted a significant difference among groups on the ‘signature change score’ (χ^2^ = 24.6, *p* < 0.001). Post-hoc pairwise comparisons indicated a significant difference between both AD (mean = 35.6% ± 32.07%) and HC (mean = 16.9% ± 24.5%; W = -3.98; p = 0.01), and between FTD (mean = 43.9% ± 34.4%) and HC (W =  5.04; p = 0.001), showing that raters were more likely to detect a signature change in clinical groups than in HC. There was no difference between AD and FTD patients (W = 1.73; *p* = 0.61).

Correlation analyses did not show any significant relationships between the ‘signature change score’ and AD and FTD clinical-demographic characteristics (i.e., age, sex, education, the time between the two signatures, MMSE raw score; all *ps* > 0.18). No significant partial correlations emerged when considering the relation between the ‘signature change score’ and both age and the time elapsed between the two signatures (*p* > 0.4).

Further Spearman correlations were run separately for neurological patients with a pathological MMSE score (< 23.80 cut-off, 62 patients, i.e., 60.2% of the clinical sample) and those with a normal MMSE score (41 patients, 39.8% of the clinical sample): no association emerged in either the first group (r = 0.07, p = 0.6), or in the second (r = 0.16, p = 0.3). Furthermore, by means an independent two-sample t-test, we did not find a significant difference between these two groups (i.e., with or without a pathological MMSE score) with respect to the signature change score (t = 1.19; p = 0.24).

Finally, the overall level of agreement among raters in the clinical groups was 39% (i.e., 39% for AD, as well as 39% for FTD). For HC, the level of agreement among raters was 58%.

**Fig. 3:**
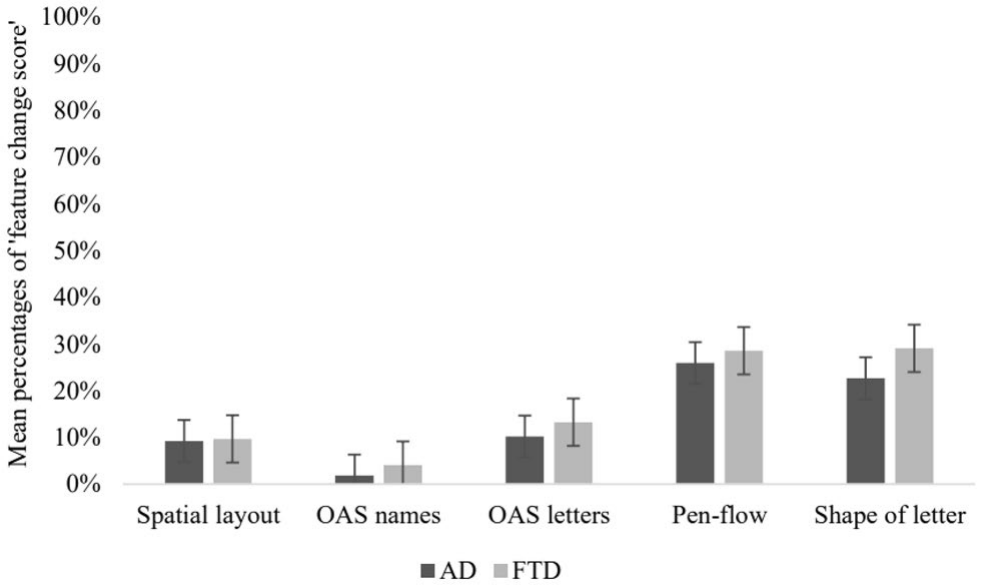
‘Feature change score’ in the experimental groups. The graph illustrates the percentage of change of different aspects of the signature in participants with Alzheimer’s diseases (AD, dark grey bars) or frontotemporal dementia (FTD, light grey bars). Error bars represent the standard error of the mean; OAS = omitted *and/or* added *and/or* switched

Qualitative changes on signature features were observable in the two clinical groups, as shown in Fig. 3. However, Kruskal–Wallis tests did not show significant differences among AD and FTD patients with respect to the signature features considered, namely: the spatial layout (χ^2^ = 0.01, *p* < 0.93), omitted/added/switched letters (χ^2^ = 1.28, *p* < 0.26), omitted/added/switched names (χ^2^ = 79, *p* < 0.37), the shape of letter (χ^2^ = 1.12, *p* < 0.29), and the pen-flow (χ^2^ = 0.07, *p* < 0.8). Additionally, correlation analyses did not show significant associations between the ‘feature change score’ and clinical and demographic factors (*e.g*., age, sex, education, the time between the two signatures, MMSE raw score; all *ps* > 0.1).

## Discussion

In the present work, we investigated possible signature changes in a large sample of participants diagnosed with dementia at different stages, by means of a rater-based approach [5]. The signature evaluation procedure was a modified version of that of Renier and colleagues [5], changing it by: 1. decreasing the ‘signature change score’ from 3 to 2 levels (0–1 score, i.e. similar vs. different signatures), hence focusing on the presence vs. the absence of change of signing; 2. exploring the change in both the overall signature as well as in some of its specific features (i.e., the spatial layout, changes of letters and names, the shape of letters, the pen-flow); 3. comparing the ‘signature change score’ within clinical groups as well as with respect to neurologically healthy controls.

Our findings are in line with the current literature, by evidencing the weak impact of cognitive decline on signing. Indeed, a minor signing change has been reported by the evaluators among both AD (36%) and FTD (44%) patients, meaning that less than half (50% of signature change score) of the evaluators detected a change in patients with dementia. In contrast to this finding, it is well-known that spontaneous writing is far more influenced by the neurodegenerative progression from the early stages of cognitive decline [11–14].

Furthermore, we did not find any significant correlation between the ‘signature change score’ and the clinical and demographic factors; this result suggests an independence of signing ability from the cognitive level of functioning, age and schooling, at least in AD and FTD.

Also, at the level of signature feature analysis, AD and FTD participants did not differ each other and were comparable to neurologically healthy persons. Although not in a statistically significant way, some effects are observable that might deserve some reflections. The signature features that appear to observers to change the most were the pen-flow (29%) and the shape of the letter (29%), while the others—the spatial layout (10%), omission, addition or exchange of letters (3%) or of names (16%)—are not reported as particularly different. The change of the pen-flow could be explained by the retrospective collection of the signatures, which cannot ensure an adequate, online, control of motor factors. Pen-flow, and to some extend even the letter shape, are connoted by high variability of signing being sensitive to contextual factors, such as the available writing space (*e.g.,* in the present study, the signature on the identity card was compared to of the informed-consent document, which is typically on a larger A4 sheet of paper), the type of pen and its ink. ‘Circumstantial’ factors [24] are particularly relevant for pen-flow, as shown by Pirlo and colleagues [20], who carried out a preliminary investigation on the features of signatures made on constrained areas, such as documents with different shapes and with variable extension of the writing spaces. On the other hand, the change in the shape of the letters should not surprise: neurodegenerative disorders may often cause central dysgraphia [25], in which the impairment of the graphemic buffer (meant as the storage of a series of graphemic units during the act of writing) may result also in deterioration of the graphemic representation [26].

The present study also highlights a quite low agreement among raters in the evaluation of the signature’s changes in both the clinical groups (39% of inter-rater agreement for both AD and FTD), as well as in neurologically healthy people (58%). These results underline the weakness of using a qualitative, rater-based, approach for determining signature changes [5], which may lead to biased and unreliable evaluations of the signature. Future studies require the use of an objective and controlled method of the signature evaluation, adding quantitative measurements of the graphic pattern to its qualitive analysis (see e.g., [19, 27–29]). Moreover, prospective studies will benefit from the collection of multiple samples of the signature at a given timepoint in order to taking into account within-subject variability [7], also deepening possible links between changes of signing and of spontaneous writing, which in the present study could not be considered due to the retrospectively nature of the data collection.

With respect to this last point, it is worth mentioning the retrospective nature of the present study that represents its major limit. However, although retrospective studies have several disadvantages (e.g., uncontrolled outcome – here the signature—assessment, selection and recall biases, difficulty in determining cause-and-effect relationship), they still provide helpful information for providing preliminary data, identifying feasibility issues and planning future prospective studies [30].

To conclude, in light of these results and remarks, we provide some further support for the view of low, maybe completely absent, vulnerability of the handwritten signature to neurodegenerative disease. The ability to sign may be preserved in dementia, at least if mild or moderate degree, and it may dissociate from the progressive cognitive decline that characterizes it, at least in the judgment of outside observers. This finding may be of relevance for the forensic practice, given the frequent necessity to qualitatively evaluate signature changes in some civil proceeding (such as the testamentary capacity assessment, see [10]). In clinical settings, the significance of signature changes requires a more in-depth, structured, assessment which may be useful to detect subclinical changes, undetectable with an observer-based approach.

## Data Availability

Data analysed in the present study are available on the Open Science Framework (OSF) repository (https://osf.io/d2vxa/).
